# Supplementary material for: A Strong Link Between Oceanographic Conditions and Zooplankton δ13C and δ15N Values in the San Jorge Gulf, Argentina
Source: Biology (Basel). 2024 Nov 29;13(12):990. doi: 10.3390/biology13120990 (PMC11727027; doi:10.3390/biology13120990)
Supplement: Supplementary file 1 [file biology-13-00990-s001.zip › biology-3318239-supplementary.pdf]

Supplementary Material

A Strong Link Between Oceanographic Conditions and Zooplankton δ13C and δ15N Values in the San Jorge Gulf, Argentina

David Edgardo Galván [1,*], Manuela Funes [2], Flavio Emiliano Paparazzo [1,3], Virginia Alonso Roldán [4,5], Carla Derisio [6], Juan Pablo Pisoni [1], Brenda Temperoni [2,6], Daniela Alejandra del Valle [6,7], Valeria Segura [6] and Seth D. Newsome [8]

[1] Centro para el Estudio de Sistemas Marinos (CESIMAR-CONICET), Boulevard Brown 2915, Puerto Madryn U9120ACD, Argentina; paparazzocnp@gmail.com (F.E.P.); pisonijp@gmail.com (J.P.P.)

[2] Instituto de Investigaciones Marinas y Costeras (IIMyC, UNMdP-CONICET), Juan B. Justo 2550, Mar del Plata B7608FBY, Argentina; manufunes15@gmail.com (M.F.); btemperoni@inidep.edu.ar (B.T.)

[3] Instituto Patagónico del Mar (IPAM-UNPSJB), Boulevard Brown 3051, Puerto Madryn U9120ACD, Argentina

[4] Universidad Tecnológica Nacional Facultad Regional Chubut, Grupo de Investigación en Gestión Desarrollo Territorial y Ambiente (GesDTA-UTNFRCH) Av. del Trabajo 1536, Puerto Madryn U9120QGQ, Argentina; virginia.a.roldan@gmail.com

[5] Instituto Patagónico para el Estudio de los Ecosistemas Continentales (IPEEC-CONICET), Boulevard Brown 2915, Puerto Madryn U9120ACD, Argentina

[6] Instituto Nacional de Investigación y Desarrollo Pesquero, Paseo Victoria Ocampo Nº1, Mar del Plata B7602HSA, Argentina; cderisio@inidep.edu.ar (C.D.); ddelvalle@inidep.edu.ar (D.A.d.V.); vsegura@inidep.edu.ar (V.S.)

[7] Consejo Nacional de Investigaciones Científicas y Técnicas (CONICET), Buenos Aires C1425FQB, Argentina

[8] Biology Department, University of New Mexico, Albuquerque, NM 87131-0001, USA; newsome@unm.edu

* Correspondence: galvan@cenpat-conicet.gob.ar; Tel.: +54-(280)-488-3184 (ext. 1277); Fax: +54-(280)-488-3543

Figure S1. Maps showing the horizontal distribution of satellite-derived chlorophyll concentrations from October 7 to December 12 in 2016 and from September 14 to November 16 in 2017. The satellite images represent 8-day averages obtained from the AquaMODIS satellite sensor, with a spatial resolution of 4 km and Level 3 processing. This processing was conducted by the Remote Sensing Program of INIDEP, using satellite data downloaded from NASA Ocean Data (https://oceandata.sci.gsfc.nasa.gov) at the NASA Goddard Space Flight Center, Ocean Biology Processing Group (2022): Moderate Resolution Imaging Spectroradiometer (MODIS) Ocean Color Data, NASA OB.DAAC, Greenbelt, MD, USA. The red rectangle highlights the conditions during the research cruise.

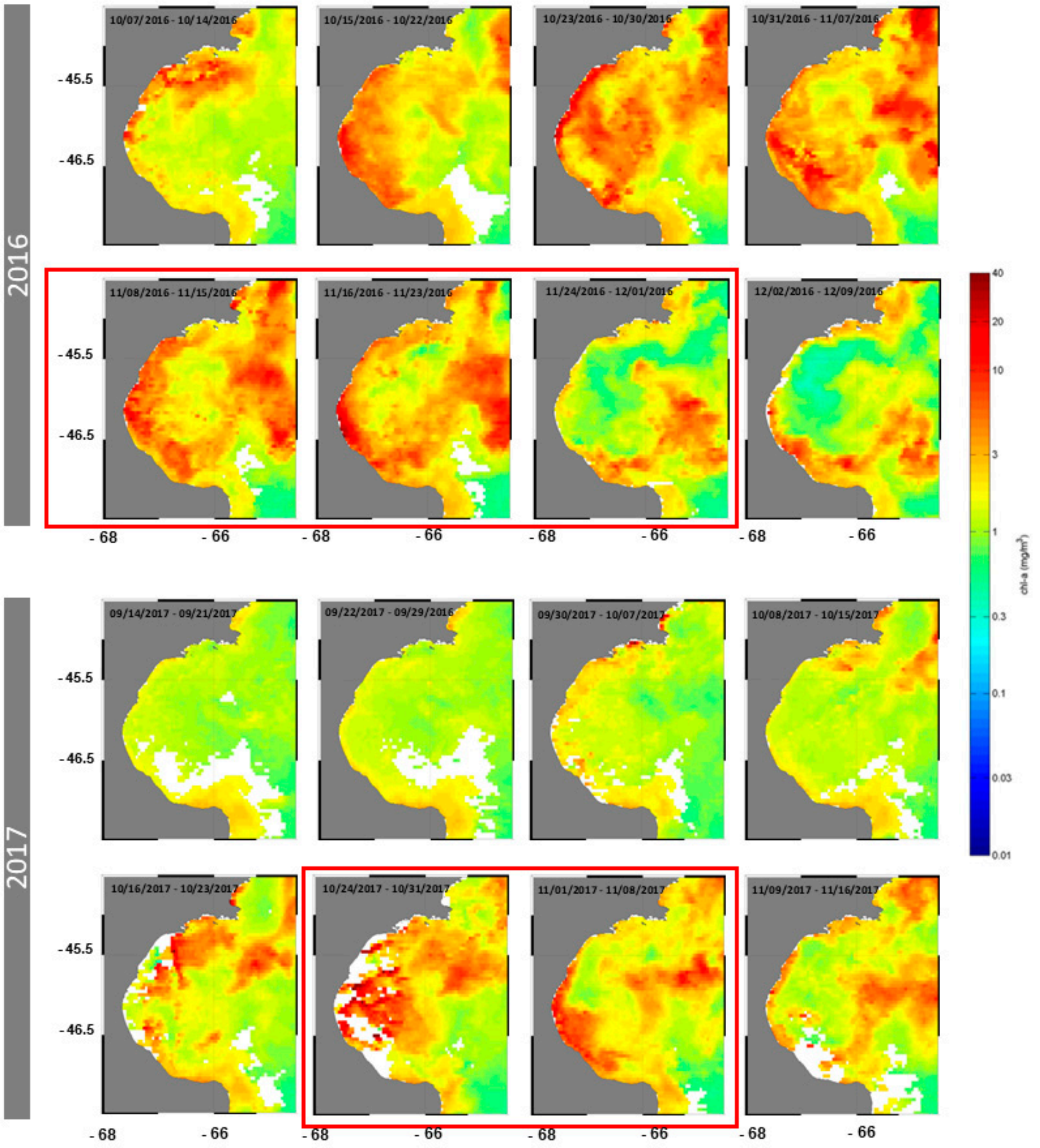

Figure S2. Plots to evaluate goodness of fit and deviations from model assumptions by visually inspection of the model δ15N ~ Stability + Surface nitrate + Surface silicic acid

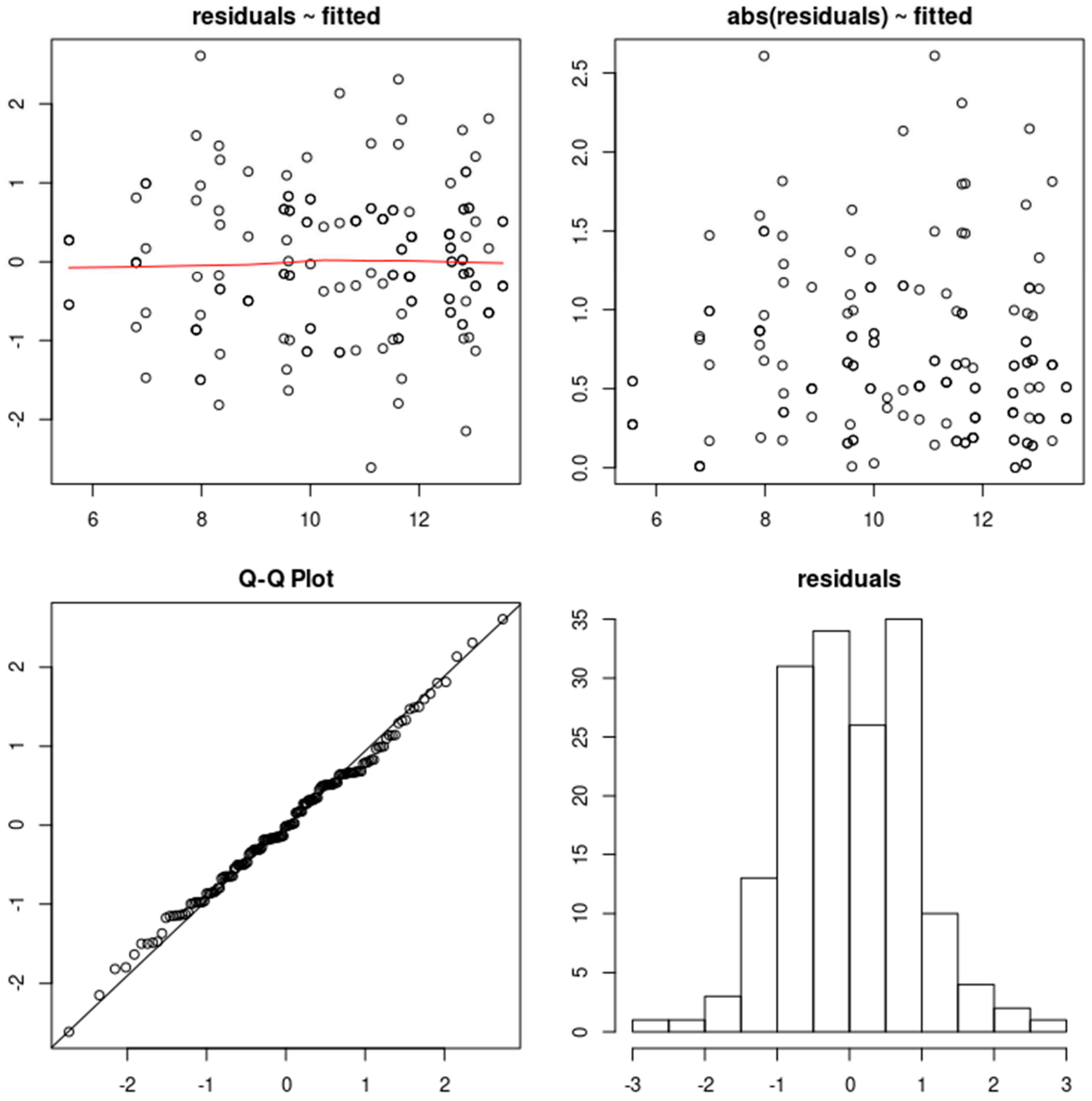

Figure S3. Plots to evaluate goodness of fit and deviations from model assumptions by visually inspection of the model δ^{13}C normalized ~ Stability + Depth + Surface nitrate

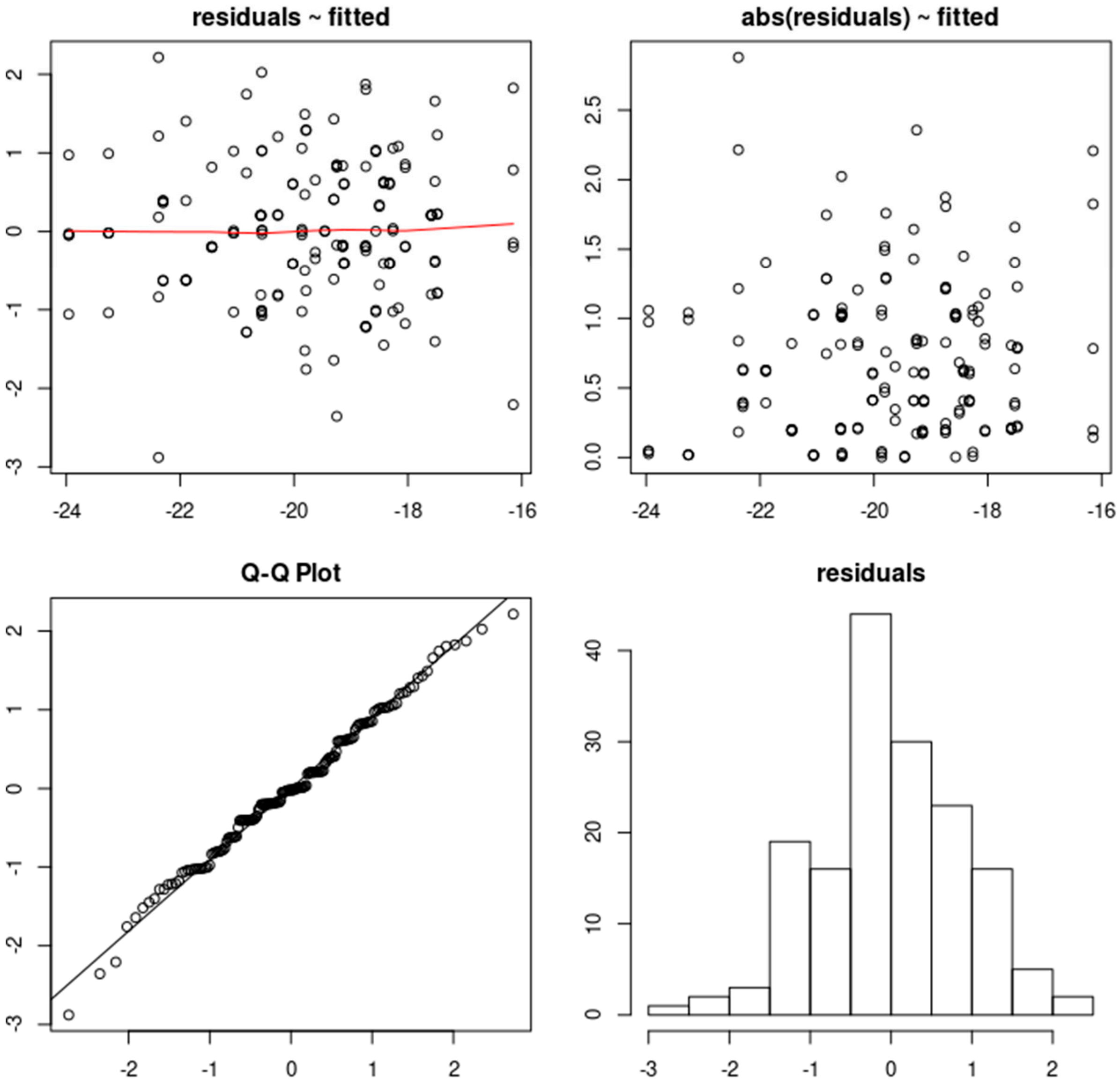

Table S1. Table showing the location of the stations in the grid sampling design. The table also shows the depth of each station and the mean $\delta^{13}C$ and $\delta^{15}N$ values observed.

ID (Station)	Year	Date	Lat.	Long.	Depth	$\delta^{15}N$ (mean)	$\delta^{13}C$ (mean)
1	2016	11/12/2016	-47.245	-65.36	55	9.5	-18.3
10	2016	11/16/2016	-46.58	-66.56	90	12.5	-18.0
12	2016	11/13/2016	-46.60	-65.82	89	10.5	-19.1
15	2016	11/17/2016	-46.22	-65.09	83	10.0	-19.2
18	2016	11/18/2016	-46.23	-66.22	101	12.5	-20.2
21	2016	11/18/2016	-46.26	-67.23	89	11.8	-17.4
22	2016	11/18/2016	-45.902	-67.30	76	12.8	-19.8
24	2016	11/19/2016	-45.90	-66.52	94	13.0	-23.9
26	2016	11/19/2016	-45.90	-65.78	100	12.6	-19.4
29	2016	11/19/2016	-45.55	-65.11	82	11.6	-17.5
3	2016	11/12/2016	-46.88	-65.11	90	5.5	-21.9
32	2016	11/21/2016	-45.56	-66.20	93	12.8	-22.3
34	2016	11/21/2016	-45.55	-66.95	86	12.9	-18.7
35	2016	11/21/2016	-45.29	-66.53	81	13.5	-18.4
37	2016	11/20/2016	-45.22	-65.75	91	13.2	-19.1
42	2016	11/22/2016	-44.91	-65.14	76	11.8	-17.5
44	2016	11/23/2016	-44.70	-65.41	79	12.8	-19.5
45	2016	11/23/2016	-44.37	-65.01	76	11.6	-18.5
5	2016	11/13/2016	-46.89	-65.87	39	9.9	-18.7
7	2016	11/14/2016	-46.90	-66.55	47	11.5	-16.1
8	2016	11/14/2016	-46.55	-67.24	79	10.2	-18.1
10	2017	11/7/2017	-46.56	-66.58	94	9.5	-19.8
12	2017	11/8/2017	-46.57	-65.82	90	9.6	-20.6
14	2017	11/5/2017	-46.57	-65.12	107	6.9	-21.2
18	2017	11/4/2017	-46.21	-66.21	102	7.9	-20.8
22	2017	11/3/2017	-45.89	-67.28	90	8.8	-20.3
24	2017	11/3/2017	-45.90	-66.57	96	9.6	-21.4
28	2017	11/3/2017	-45.90	-65.11	79	6.8	-22.9
32	2017	11/2/2017	-45.57	-66.19	92	7.9	-21.8
35	2017	11/1/2017	-45.21	-66.50	81	7.9	-20.9
37	2017	11/1/2017	-45.22	-65.81	91	10.8	-20.5
39	2017	10/31/2017	-45.23	-65.10	88	11.1	-18.7
43	2017	10/31/2017	-44.91	-65.45	82	11.3	-19.3
5	2017	11/6/2017	-46.91	-65.82	37	8.3	-19.5
8	2017	11/6/2017	-46.53	-67.30	76	8.34	-20.2

Table S2. Rounds of model fitting to select the best random structure. Models were fitted using the lme and gls functions of the nlme package [53]. At this stage, models were fitted by maximizing the restricted log-likelihood [49].

Response variable	Fixed factors	Random factors	AIC
$\delta^{15}N$	Stability + Depth + Surface nitrate + Surface silicic acid	-	564
		Year	516
		Station	426
		Year/Station	18
$\delta^{13}C$ normalized	Stability + Depth + Surface nitrate + Surface silicic acid	-	582
		Year	579
		Station	454
		Year/Station	-26

Table S3. Linear models of the residuals and absolute values of the model residuals against the predicted values to explore departures from homogeneity of variance [49].

Response variable	Fixed factors	Test	P (b= 0)
$\delta^{15}N$	Stability + Surface nitrate + Surface silicic acid	residuals ~ fitted	0.85
		abs(residuals) ~ fitted	0.35
$\delta^{13}C$ normalized	Stability + Depth + Surface nitrate	residuals ~ fitted	0.86
		abs(residuals) ~ fitted	0.47
